# A Review of Mechanical Performance Studies on Composite Concrete Beams and Slabs

**DOI:** 10.3390/ma18143259

**Published:** 2025-07-10

**Authors:** Xinhao Wang, Qiuwei Yang, Xi Peng, Kangshuo Xia, Bin Xu

**Affiliations:** 1 School of Civil and Transportation Engineering, Ningbo University of Technology, Ningbo 315211, China; wangxinhao0709@nbut.edu.cn; 2 Engineering Research Center of Industrial Construction in Civil Engineering of Zhejiang, Ningbo University of Technology, Ningbo 315211, China; 3 Ningbo Roaby Technology Industrial Group Co., Ltd., Ningbo 315800, China; 5632374376@aliyun.com; 4 Key Laboratory of New Technology for Construction of Cities in Mountain Area, School of Civil Engineering, Chongqing University, Chongqing 400045, China; xiakangshuo@cqu.edu.cn

**Keywords:** UHPC, ECC, RAC, composite beam, composite slab

## Abstract

This paper reviews the applications and performance advantages of ultra-high-performance concrete (UHPC), engineered cementitious composite (ECC), and recycled aggregate concrete (RAC) in composite flexural members. UHPC is characterized by its ultra-high strength, high toughness, excellent durability, and microcrack self-healing capability, albeit with high costs and complex production processes. ECC demonstrates superior tensile, flexural, and compressive strength and durability, yet it exhibits a lower elastic modulus and greater drying shrinkage strain. RAC, as an eco-friendly concrete, offers cost-effectiveness and environmental benefits, although it poses certain performance challenges. The focus of this review is on how to enhance the load-bearing capacity of composite beams or slabs by modifying the interface roughness, adjusting the thickness of the ECC or UHPC layer, and altering the cross-sectional form. The integration of diverse concrete materials improves the performance of beam and slab elements while managing costs. For instance, increasing the thickness of the UHPC or ECC layer typically enhances the load-bearing capacity of composite beams or plates by approximately 10% to 40%. Increasing the roughness of the interface can significantly improve the interfacial bond strength and further augment the ultimate load-bearing capacity of composite components. Moreover, the optimized design of material mix proportions and cross-sectional shapes can also contribute to enhancing the load-bearing capacity, crack resistance, and ductility of composite components. Nevertheless, challenges persist in engineering applications, such as the scarcity of long-term monitoring data on durability, fatigue performance, and creep effects. Additionally, existing design codes inadequately address the nonlinear behavior of multi-material composite structures, necessitating further refinement of design theories.

## 1. Introduction

In China’s economic structure, the construction industry holds a pivotal position as one of the cornerstone industries, directly influencing the overall growth of the national economy. With the robust development of the construction industry, concrete, as the core structural material, has witnessed a surge in consumption over the past two decades. In 2023, the usage of commercial concrete in China reached a staggering 3.293 billion cubic meters [1]. The production process of concrete is accompanied by significant CO_2_ emissions and energy consumption, posing severe challenges to resources, energy, and the ecological environment [2]. To align with the new societal demands for “green, low-carbon, and high-quality” development, the construction industry has reached a consensus on vigorously promoting energy-efficient and environmentally friendly buildings, a cause that has garnered significant attention from both academic and engineering circles [3]. Dating back to the 1960s, bridge construction in Europe, America, and Japan entered a golden age, where composite structures emerged with their unique advantages. This structural form not only optimized the overall mechanical performance economically but also skillfully integrated the strengths of various materials, such as the strong tensile strength of steel [4] and the excellent compressive performance of concrete [5], demonstrating the rationality of material application. Meanwhile, the construction convenience of composite structures greatly enhanced construction efficiency [6], meeting the dual demands for speed and quality in bridge construction at that time, and thus was widely adopted. Therefore, in the face of the urgent need for the sustainable development of concrete materials, it is imperative for us to draw on the successful experiences of composite structures in history. Through technological innovation, we aim to reduce carbon emissions and energy consumption during concrete production, enhance the environmental performance and service life of materials, and promote the optimization of structural design, thereby achieving efficient resource utilization and harmonious coexistence with the ecological environment.

The term “composite structure” refers to a structure composed of composite structural members, as well as a structure composed of composite structural members combined with steel members or RC members. A composite structural member is a structural member that can bear loads as a whole through the combination of steel or other non-cement-based materials with RC. This combination integrates concrete with materials that have significantly different properties from concrete (such as steel, FRP materials, etc.) [7]. In contrast, “composite concrete structure” involves the combination of cement-based materials. Compared to combinations of concrete with other materials, the combination of different concretes exhibits greater compatibility. With appropriate treatment, the interface between different concretes can achieve excellent integrity [8]. Concrete is primarily combined in the following three aspects [9]:

(1) Material level: Concrete preparation involves the use of different materials, including aggregates (such as natural aggregates, recycled aggregates, lightweight aggregates, metal aggregates), functional materials, and admixtures.
(2) Component level: Concrete is combined at the sectional and longitudinal levels of the component. At the component section, different strengths or functional concretes are combined to form gradients or layers. The combination types include strength (high-strength, low-strength concrete), type (recycled concrete, seawater sea-sand concrete), composition (fiber, rubber), function (waterproof, fire-resistant, sound insulation), etc. At the longitudinal level of the component, different concretes are combined in segments according to the stress zone (such as the plastic hinge zone and the middle section of a beam) or the constraint conditions (areas with different deformation and rotation requirements).
(3) Structural level: Integrating different concrete components by strategically placing the most suitable members where they are most required to meet both structural demands and functional requirements.

In the field of structural engineering, beams, slabs, and columns—the three core components of load-bearing systems—have consistently been the focal points of research and technological innovation in structural mechanical performance. These components collaborate to form the spatial load transmission network of buildings: beams, as horizontal load-bearing elements, undertake the tasks of load transfer under bending moments and shear forces; slabs, acting as bidirectional planar members, facilitate the lateral distribution of vertical loads and serve as spatial interface elements [10]; columns, as vertical load-transmission hubs, efficiently transfer upper-level loads to the foundation system [11]. In recent years, driven by the development of super-tall buildings, large-span spatial structures, and complex loading environments, research has established a comprehensive technical framework encompassing design, construction, and whole-life monitoring, leveraging numerical simulation and intelligent monitoring technologies. This advancement has promoted the engineering application of composite structures and high-performance materials, achieving the synergistic optimization of structural safety, economy, and functionality. This paper reviews experimental research and numerical analyses of composite concrete structures in beam-slab flexural members, aiming to provide support for the development of composite concrete components. Table 1 lists the abbreviations of the professional terms used in this paper. Currently, although research on composite concrete members is gradually increasing, a systematic review in this field remains highly necessary. This review conducts a literature search on keywords such as “UHPC”, “ECC”, “RAC”, “composite beam”, and “composite slab” based on the Scopus database and the China National Knowledge Infrastructure (CNKI) database. The review primarily focuses on the mechanical properties of composite concrete beams and slabs, serving as both a summary of existing research literature and offering insights into the future research priorities for composite components.

## 2. Materials of Composite Concrete Structures

In the field of contemporary civil engineering, innovations in materials science are redefining the performance boundaries and sustainability of architectural structures. Ultra-high-performance concrete (UHPC), engineered cementitious composite (ECC), and recycled aggregate concrete (RAC) have garnered significant attention in modern infrastructure construction due to their exceptional mechanical properties or environmental benefits. UHPC, with its exceptional compressive strength and dense microstructure, demonstrates irreplaceable advantages in projects requiring ultra-long durability and high load-bearing capacity, such as bridges, high-rise buildings, and nuclear power facilities. ECC achieves metal-like tensile strain-hardening behavior through fiber reinforcement technology, effectively controlling crack widths and significantly enhancing structural toughness under seismic or impact loads. Meanwhile, RAC contributes to green construction under the circular economy model by recycling aggregates from construction waste, maintaining fundamental mechanical performance while reducing carbon emissions by over 30%. The selection of these three materials not only reflects the engineering sector’s pursuit of extreme environmental adaptability, structural safety redundancy, and resource efficiency but also signifies a paradigm shift in materials research—from single-performance optimization to holistic lifecycle sustainability management.

### 2.1. UHPC

UHPC is composed of high-quality cement, fine aggregates, quartz sand, high-reactivity mineral admixtures, high-strength steel fibers or polymer fibers, and high-range water-reducing admixtures [12]. Recognized as one of the advanced strategies addressing modern construction challenges [13], UHPC demonstrates significant advantages in strength, durability, and workability. Compared to normal concrete (NC), UHPC achieves qualitative performance improvements, particularly exhibiting exceptional performance under complex environmental conditions and showcasing substantial potential in materials science and engineering applications. Through the elimination of coarse aggregates to enhance material homogeneity and optimization of particle gradation with refined components, UHPC achieves remarkably high compactness and strength. In this process, high-reactivity mineral admixtures represented by silica fume and slag powder play a pivotal role, significantly enhancing the mechanical properties of UHPC [14,15,16,17].

**Figure 1:**
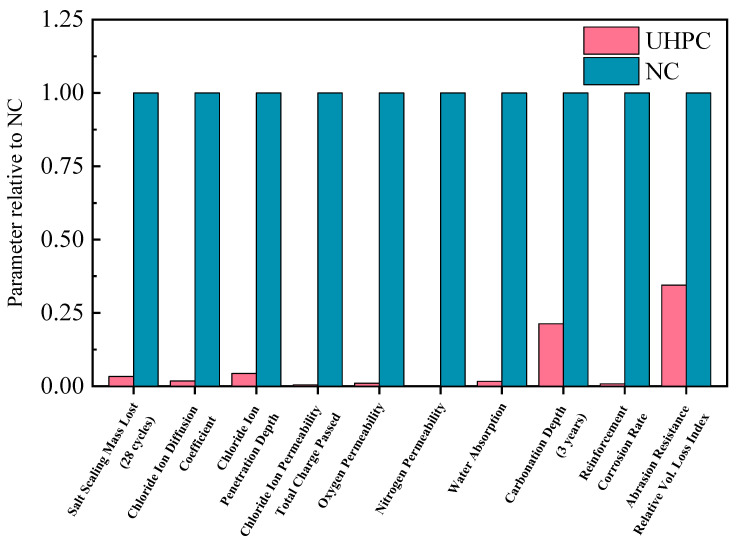
Comparison of durability performance between UHPC and NC.

UHPC exhibits excellent properties, such as ultra-high strength, high toughness, high durability, and strong self-healing ability for microcracks, which can effectively improve its service life and reduce maintenance. The compressive strength of UHPC generally exceeds 150 MPa, which is about 3 to 16 times that of NC. By incorporating steel fibers, the ductility and energy absorption capacity of UHPC usually exceed those of High-Performance Concrete (HPC) by more than 300 times, which can effectively improve the seismic performance of members [18]. The high durability of UHPC is reflected in its ability to resist environmental erosion, such as freeze–thaw resistance, salt erosion resistance, and carbonization resistance. Compared with NC, all durability indicators of UHPC are better, and the durability comparison results between NC and UHPC are listed in Figure 1 [19,20]. This benefits from UHPC’s own dense and uniform structure, extremely few pores, and very small water-binder ratio. There is almost no excess moisture inside, which helps it better resist the penetration of potentially harmful and corrosive media, such as chloride, sulfate, water, carbon dioxide, and oxygen, thus exhibiting excellent carbonization resistance, impermeability, freeze–thaw resistance, and wear resistance [21,22]. Moreover, studies have shown that the freeze–thaw scaling of UHPC is 1/140 of that of NC, indicating that UHPC has excellent freeze–thaw resistance [23] and can be used in areas with large diurnal temperature differences. Liu et al. [24] found that after 600 freeze–thaw cycles, the durability coefficient of UHPC is greater than or equal to 100, and the mass loss is almost zero. Lee et al. [25,26] confirmed the low permeability and freeze–thaw resistance of UHPC, indicating its excellent durability in different applications. Despite its many advantages, UHPC also has relatively prominent disadvantages. The cement content of UHPC is as high as 800–1000 kg/m^3^, and it has a large hydration heat and a more complex production process, is prone to shrinkage cracks, and has a high cost [27].

### 2.2. ECC

ECC was first successfully developed in the United States. Subsequently, Japan and European countries conducted research on ECC and proposed related concepts, such as Ductile Fiber-Reinforced Cementitious Composites (DFRCCs) and Strain-Hardening Cement-Based Composites (SHCCs). In 2000, Xu Shilang’s team conducted in-depth research on this material and named it Ultra-High Toughness Cementitious Composites (UHTCCs) [28]. The components of ECC include water, cement, fly ash, quartz sand, fibers, chemical additives, etc. Compared to concrete, ECC exhibits four major advantages [29,30,31,32]:

Firstly, it exhibits excellent tensile properties. Relevant studies indicate that the ultimate tensile strain of ECC far exceeds that of ordinary concrete, reaching over 3%, which is tens to hundreds of times higher than conventional concrete [33,34,35].

Secondly, it demonstrates superior flexural performance. During structural service, ECC exhibits a steady-state cracking mode with multiple microcracks. The crack width under saturated cracking conditions remains below 0.1 mm, and it shows excellent compressive toughness as well as deformation compatibility with steel reinforcement. Its flexural deflection capacity is also several times greater than that of conventional concrete.

Thirdly, it possesses favorable compressive properties. Due to the fiber bridging effect within the matrix, ECC achieves significantly higher compressive strength than ordinary concrete. Under ultimate load failure, it maintains structural integrity without crushing collapse, unlike conventional concrete.

Fourthly, it offers enhanced durability, primarily characterized by excellent impermeability. Experimental studies show that after hundreds of freeze–thaw cycles, the ultimate tensile strain of ECC decreases by less than 2%, with no significant degradation in material performance.

However, despite these excellent properties that make ECC suitable for architectural applications, it also has certain drawbacks. Firstly, due to the absence of coarse aggregates and its higher cement and polymer fiber content [36], the elastic modulus of ECC is lower than that of ordinary concrete [37]. Secondly, the elevated cementitious material content in ECC results in higher drying shrinkage strains [38].

### 2.3. RAC

RAC is a novel green concrete produced by artificially processing waste concrete into recycled aggregates to replace natural aggregates [39,40]. RAC addresses both the shortage of sand and gravel resources and the environmental hazards posed by construction waste, aligning with China’s dual carbon goals and sustainable development strategy. However, despite its significant environmental benefits, RAC faces technical and performance challenges in practical applications. Since RAC coarse aggregates are typically derived from crushed waste concrete elements, they are often coated with residual cement mortar, exhibit angular shapes, and contain internal microcracks from the crushing process. These characteristics place recycled coarse aggregates at a disadvantage compared to natural aggregates, manifesting as higher crushing indices, lower density, and greater water absorption—all of which adversely affect structural safety [41,42,43,44,45].

With continuous advancements in research methodologies and technologies, certain RAC formulations now demonstrate mechanical properties that match or exceed those of conventional concrete. For instance, RAC utilizing high-quality processed recycled aggregates can achieve 95–100% of the compressive strength of ordinary concrete [46]. Some RAC variants exhibit superior tensile and flexural toughness, with crack resistance improved by approximately 10–15% [47]. Under dynamic load conditions, RAC may even demonstrate equivalent or superior performance compared to conventional concrete [48,49].

In summary, this section has reviewed the research status of several common cement-based materials. UHPC, ECC, and RAC serve as composite structural materials with distinct characteristics, each demonstrating significant potential across diverse applications. UHPC, characterized by its ultra-high strength, exceptional toughness, superior durability, and robust self-healing capacity for microcracks, has emerged as a cutting-edge solution for addressing construction challenges under complex environmental conditions. ECC, with its outstanding tensile, flexural, and compressive performance, as well as its durability, exhibits broad application prospects in the construction industry. Meanwhile, RAC, as a green concrete, not only mitigates the shortage of sand and gravel resources but also effectively addresses construction waste management, aligning with sustainable development strategies. However, each of these materials presents certain limitations: UHPC involves high costs; ECC exhibits relatively low elastic modulus and elevated drying shrinkage strains; and RAC faces technical and performance-related challenges that require resolution.

UHPC, ECC, RAC, and NC each exhibit distinct advantages and disadvantages across various performance metrics. In practical engineering applications, the selection of the most suitable concrete material should be based on a comprehensive evaluation of project requirements, environmental conditions, economic feasibility, and other relevant factors. To provide a clearer overview of their performance characteristics, Table 2 presents selected key performance indicators for UHPC, ECC, and NC [50,51,52].

## 3. Performance of Composite Concrete Elements

### 3.1. UHPC Composite Components

Leveraging its ultra-high strength, exceptional toughness, and durability, UHPC has demonstrated significant advantages in composite structural applications in recent years. However, the high cost of UHPC material poses economic challenges when used for entire structural components. To maximize the mechanical and durability benefits of UHPC while minimizing construction expenses, researchers have proposed hybridizing UHPC with NC to form composite structures. This approach not only addresses the limitations of NC—such as low load-bearing capacity, poor ductility, and inadequate durability—but also effectively utilizes the superior mechanical properties and longevity of UHPC. Furthermore, this hybrid solution offers construction convenience and cost-effectiveness advantages [53,54,55].

For UHPC composite beam members, the application of UHPC significantly enhances structural mechanical responses in terms of flexural performance improvement. Experiments demonstrate that UHPC reinforcement can double the load-bearing capacity of original beams while maintaining high load levels, even during large deflection stages [56]. Positioning the UHPC layer at the beam’s bottom effectively restricts crack propagation and enhances ultimate load-carrying capacity [57]. Furthermore, prestressed-UHPC composite reinforcement technology elevates the crack resistance of reinforced concrete (RC) beams by 50–80%, while optimizing stress distribution to mitigate deformation in tensile zones [58]. Singh et al. [59], through combined experimental and numerical analyses, revealed that reinforced UHPC reinforcement can enhance beam crack resistance by 3.5 times, underscoring its crack-mitigation efficacy. Regarding parameter influences, Wang et al. [60] systematically elucidated the mechanisms by which UHPC layer height and cross-sectional configuration affect flexural behavior, establishing a computational model for ultimate bending moment prediction. Research by Li et al. [61,62] demonstrated the exceptional performance of prestressed UHPC-NC trough composite beams in negative moment regions, confirming a strong positive correlation between prestress reinforcement ratios and both cracking loads and ultimate load capacities. Numerous scholars have conducted research on the shear properties of UHPC-NC and NC-NC bonding, as shown in Table 3 [63,64,65,66,67].

Based on extensive experimental research, scholars have proposed a series of design theories and computational methods. Studies indicate that UHPC-NC composite interfaces exhibit excellent cooperative behavior. Liang et al. [68] confirmed through flexural tests that no slippage or spalling occurred at the composite interface, leading to the development of a formula for calculating the flexural capacity of RC beams with non-removable formwork. Bahraq et al. [69,70] further validated that the composite structures adhere to the plane section assumption, providing foundational support for theoretical analyses. Li et al. [61,62] conducted static tests to investigate the flexural performance in negative moment regions, obtaining critical experimental data including deformation characteristics, cracking loads, crack distribution patterns, failure loads, and strain profiles. Leveraging these experimental findings and existing research outcomes, they derived computational models for calculating the initial cracking moment and flexural capacity of both the inner NC core and the UHPC outer layer within composite beams:

$$\begin{array}{l} \int_{cc}\sigma_{cc}bdx+\int_{uc}\sigma_{uc}bdx=\int_{ut}\sigma_{ut}bdx+A_{p}f_{p}+A_{s}f\\ M_{u}=\int \sigma_{c}y_{c}bdx+\int \sigma_{t}y_{t}bdx+A_{p}f_{p}y_{p}+A_{s}f_{y}y_{s} \end{array},$$

In the formula, b represents the cross-sectional width; $\sigma_{cc}$ represents NC compressive stress; $\sigma_{uc}$ represents the compressive stress of UHPC; $\sigma_{ut}$ represents the tensile stress of UHPC; $A_{p}$ represents the cross-sectional area of prestressed steel bars; and $A_{s}$ represents the cross-sectional area of ordinary steel bars.

For UHPC composite slab members, compared to composite beam structures, the low shear-span ratio characteristic of slabs results in lower interfacial shear stresses under identical deflection conditions. Integrating UHPC into conventional slab systems to form UHPC-NC composite configurations leverages UHPC’s high tensile strength and strain-hardening properties, significantly enhancing structural crack resistance and durability [71]. This approach provides enhanced safety margins for interface design.

Nie et al. [72] conducted experiments on four groups of simply supported high-strength concrete (HSC) composite slabs to systematically investigate the influence of various interface treatment methods on shear performance. The loading method is single-point monotonic static loading at the midspan. The results demonstrated that conventional approaches (including natural vibration, surface troweling, roughening, and shear reinforcement configuration) effectively prevented shear failure at the composite interface prior to flexural failure.

Wan [73] and Wang [74] enhanced interface bonding through the incorporation of truss reinforcement and stirrups, conducting flexural performance tests along with ductility and stiffness assessments on UHPC composite slabs with different composite configurations. All adopt the reverse loading form, applying vertical load at the midpoint of the span to form a pure bending section. Their research confirmed that conventional interface treatments fully satisfy the shear resistance requirements of UHPC composite slabs. It was also observed that UHPC composite slabs reinforced with truss and stirrups exhibited higher early-stage flexural stiffness and load-bearing capacity, albeit with reduced displacement ductility and energy dissipation ductility.

Li [75] performed four-point bending tests to analyze the effects of critical parameters such as UHPC layer thickness ratio, concrete strength, steel fiber content, and reinforcement ratio. The experimental method is four-point bending loading. The study revealed a linear correlation between cracking load, ultimate bearing capacity, and increasing UHPC layer thickness ratios, while concrete strength and steel fiber content demonstrated negligible impacts on mechanical properties. Notably, reinforcement within the UHPC layer significantly improved failure mode characteristics.

Wang et al. [76] analyzed various key factors affecting the performance of composite plates through finite element numerical analysis and verification. The results indicate that the use of UHPC significantly improves the bending performance of composite panels, and they propose a set of bending capacity calculation formulas suitable for UHPC-NC prefabricated hollow composite panels.

Relevant studies indicate that the failure modes of UHPC-NC composite slabs resemble those of conventional NC composite slabs [77]. During loading, the interfacial bonding between UHPC and RC enables collaborative stress transfer [78,79]. However, when the UHPC substrate is relatively thin, interfacial bonding performance weakens, leading to reduced flexural capacity [80]. Increasing the UHPC-to-RC layer thickness ratio enhances both ultimate bearing capacity and cracking load, with the initial crack location dependent on UHPC thickness. Longitudinal reinforcement ratio exerts the most significant influence on flexural capacity [77,81]. Table 4 summarizes key experimental designs, data, and conclusions from selected literature on UHPC composite components.

By leveraging the synergistic interaction between UHPC and conventional concrete, UHPC composite structures achieve a dual optimization of performance and cost—retaining UHPC’s ultra-high strength, exceptional toughness, and durability while significantly reducing overall material expenses. In terms of parametric design theory, quantitative models based on parameters such as UHPC layer thickness ratio and reinforcement ratio have been established. These models clarify the coupled effects of UHPC layer height and cross-sectional configuration, as well as the dominant impact of reinforcement ratio on cracking loads. Nevertheless, challenges persist, including critical thresholds for interfacial performance, gaps in long-term performance evaluations, and limitations in design theory applicability. Future research directions may focus on lifecycle performance characterization and the development of standardized design frameworks. With advancements in computational modeling and technological innovation, the application prospects of such high-performance composite structures in bridges, buildings, and other sectors are promising, potentially driving the evolution of concrete structures toward enhanced performance and sustainability.

### 3.2. ECC Composite Components

ECC has demonstrated unique advantages in composite structural applications in recent years due to its ultra-high toughness, strain-hardening behavior, and multi-crack steady-state cracking capability. Unlike UHPC, which is renowned for ultra-high strength, ECC achieves 3–7% ultimate tensile strain with fiber contents below 2% through micromechanical design, while maintaining saturated crack widths under 0.1 mm. These properties provide distinct advantages in safety, durability, and applicability. Addressing the brittle failure, inadequate ductility, and durability limitations of traditional RC structures, researchers have proposed hybridizing ECC with RC to form innovative composite components. This breakthrough approach overcomes the bottlenecks of low load-bearing capacity and poor seismic performance in RC structures while significantly enhancing overall structural ductility through ECC’s high energy dissipation capacity.

Recent systematic advancements have been made in studying interface bonding and the structural performance of ECC composite beams. In terms of interface treatment, Qiao et al. [82], Khan et al. [83], and Cui et al. [84] conducted experimental investigations to analyze the effects of different interface treatment methods on the load-bearing capacity of ECC-NC composite beams. Building upon existing research, Shazab et al. [85], Shin et al. [86], and Gao et al. [87] performed numerical simulation analyses of ECC-NC composite beams. All studies collectively demonstrated that the ECC-NC interface exhibits excellent bonding performance without debonding failure, and the cross-sections adhere to the plane section assumption. Reference [88] summarizes the interfacial bonding strength between ECC and ordinary concrete, where the diagonal shear strength is much higher than other indicators. Similarly, Table 5 presents experimental data on the interfacial bond strength between ECC and ordinary concrete reported in several other studies in the literature. The results indicate that interface treatment methods with high roughness, such as sandblasting and grooving, can better improve the bonding strength.

Structural performance research has also progressed in flexural and shear behaviors. In terms of flexural capacity, Yin et al. [93] and Wu et al. [94] conducted experimental studies on the normal section bending behavior of ECC-NC composite beams, with variables including the thickness and geometry of the ECC layer in the tensile zone, as well as reinforcement ratios. Hu et al. [95] performed modeling analysis based on experimental results and proposed a formula for calculating the normal section flexural capacity of ECC-NC composite beams. In shear performance investigations, studies on longitudinal shear behavior in ECC-NC composite slabs through shear-span ratio modifications have been conducted by Mohammed et al. [96], while Hossain et al. [97] and He et al. [98] examined how composite layer thickness influences shear resistance. Collectively, these works demonstrate that ECC-NC composite beams achieve superior load-bearing capacity and ductility compared to conventional concrete beams—a performance advantage attributed to ECC’s enhanced strain-hardening capacity and crack-arresting properties. This benefit is particularly pronounced in monolithically cast specimens. Despite these advancements, gaps persist in understanding the mechanistic evolution of flexural performance, establishing consensus on optimal composite layer thicknesses and reinforcement ratios, and developing corresponding analytical frameworks for predictive modeling.

Compared to ECC composite beams, relatively few studies have focused on ECC composite slab systems. Liu [32] designed 100 mm × 500 mm × 2100 mm slab specimens to investigate the influence of PVA-ECC on the flexural performance of RC members. The test results revealed excellent bonding between PVA-ECC and concrete during loading, with no debonding failures observed at the material interface. PVA-ECC exhibited multi-cracking behavior that effectively suppressed cracking in the upper concrete layer, significantly enhancing the specimens’ ultimate load-bearing capacity. A formula for calculating the flexural capacity of PVA-ECC slabs was derived, providing theoretical support for research and design of ECC-concrete composite slabs.

Lin et al. [99] developed an innovative FRP SS-ECC composite slab utilizing FRP (fiber-reinforced polymer) profiles as both formwork and tensile reinforcement, with seawater sea-sand engineered cementitious composites (SS-ECCs) substituting for conventional concrete. The splicing surface was coated with the epoxy resin for bonding. Experimental investigations under four-point bending, central single-line loading, and off-center single-point loading regimes revealed distinct mechanical characteristics: while exhibiting lower ductility and reduced energy absorption capacity compared to RC counterparts, the composite slab demonstrated significantly elevated ultimate load-bearing capacity accompanied by substantial residual strength post-peak failure.

Deng et al. [100] fabricated prefabricated composite slabs using ultra-lightweight ECC, demonstrating a 29% higher load-bearing capacity and 24% lower self-weight compared to conventional NC slabs through four-point bending experiments.

Gao et al. [101] utilized hybrid-fiber ECC—known for its superior ductility—as an overlay on NC substrates to form composite pavement slabs. Investigations into the flexural fatigue properties of these hybrid-fiber ECC–concrete composite slabs, conducted through four-point bending experiments, revealed significant advantages in bending fatigue resistance compared to conventional concrete slabs, particularly as the overlay thickness increased.

Deng et al. [102] leveraged the lightweight, high-strength, and high-ductility properties of lightweight ECC to address the limitations of conventional concrete composite slabs, such as excessive self-weight and cracking susceptibility.

Kang et al. [103] proposed a method for fabricating fully encapsulated ECC–concrete composite specimens, systematically studying their basic mechanical properties through compression, tension, and flexural tests. Numerical analyses were also conducted to investigate the flexural performance of reinforced composite beams. Upon failure, the ECC–concrete interface remained free of slippage, indicating reliable bonding and collaborative stress transfer. The composite specimens exhibited enhanced compressive, tensile, and flexural strengths compared to conventional concrete specimens, with the most significant improvement observed in flexural strength.

Current research findings demonstrate that the composite application of ECC and RC significantly mitigates the brittle failure, inadequate ductility, and durability limitations of traditional RC structures. Interface experiments confirm that ECC-NC interfaces achieve reliable collaborative stress transfer through treatments such as U-shaped formwork and reinforcement connections, with no debonding observed and compliance with the plane section assumption. Structurally, ECC-NC composite beams exhibit enhanced flexural capacity with increasing ECC layer thickness and improved shear performance (40–60% enhancement at shear-span ratios of 1.5–2.5), particularly in monolithically cast members with superior combined flexural-shear behavior. For slab components, composite designs reduce self-weight while increasing load-bearing capacity and demonstrating notable fatigue resistance advantages. Although partial capacity calculation formulas have been established, the evolution of flexural performance mechanisms, optimal composite layer thicknesses, and reinforcement ratio thresholds remain undefined. Design theories have yet to fully align with material innovations, indicating a need for further standardization. Table 6 summarizes key experimental designs, data, and conclusions from selected literature on ECC composite components.

### 3.3. RAC Composite Components

RAC, an environmentally friendly and resource-efficient material, is gaining increasing attention. However, current research predominantly focuses on material properties rather than the performance of composite components. This paper reviews recent research achievements on RAC composite structures and synthesizes the findings, as summarized in Table 7.

Wang et al. [104] conducted comparative tests on NC composite beam and RAC composite beam. The loading method is two-point static loading. The results showed similar failure modes between the two beam types, with shear-compression failure observed at shear-span ratios of 1–3. The ultimate load-bearing capacity decreased with increasing shear-span ratio, and the RAC composite beam demonstrated higher capacity than the NC composite beam.

Xiao et al. [105] conducted shear tests on RAC composite beams. The bond interface of U-type beams was designed as a flat surface, while the bond interface of C-type beams featured a roughened texture created through brushing or combing treatments. The study revealed that for C-type and U-type RAC composite beams with a shear-span ratio of 3.0, their load-bearing performance was comparable to monolithic beams, demonstrating no adverse effects on either ultimate load capacity or deformation behavior under experimental conditions.

Lapko et al. [106] proposed a novel composite structure using RAC as the base material, incorporating HSC in the compressive zone of members. The loading method is four-point loading. Experimental and numerical analyses revealed that composite beams achieved 20–36% higher flexural capacity and 20–40% reduced deflection compared to monolithic RAC beams, demonstrating enhanced stiffness and bending resistance.

Fahmy et al. [107] designed composite T-shaped beams comprising an external prefabricated U-shaped high-strength NC web and a core-filled region. The core zone utilized C50 RAC or C25 RAC, while the beam flanges were constructed with C50 NC or C50 RAC. The loading method is four-point loading. Results showed that both composite beams exhibited 11.4% and 9.8% higher flexural strength, 20% and 13.5% increased yield loads, and 10–55% reduced ductility compared to reference beams. Notably, the C25 RAC-filled beam demonstrated deformation capacity and compressive strength comparable to the reference specimen.

Qin et al. [108] developed a UHPC-RAC composite section and fabricated prefabricated UHPC-RAC composite beams. The interface junction is designed with rectangular keyways. Through four-point flexural tests, they analyzed the effects of tensile UHPC layer thickness, UHPC-RAC interface roughness, and lateral UHPC height on failure mechanisms, load-bearing capacity, deformation, and initial stiffness. A formula for calculating load-bearing capacity was subsequently proposed:

$$\begin{array}{l} \alpha_{c1}f_{cc}bx_{n}=f_{ut}bh_{u}+f_{y}A_{st}\\ M_{u}=\alpha_{c1}\beta_{c1}f_{cc}bx_{n}(h-a_{s}-\frac{\beta_{c1}x_{n}}{2})+f_{ut}bh_{u}(a_{s}+\frac{1}{2}h_{u}) \end{array}$$

In the formula, h—cross-section height; b—cross-section width; $h_{u}$—thickness of UHPC on tensile side; $x_{n}$—actual height of compression zone; $a_{s}$—distance from resultant force point of tensile reinforcement to edge of tensile zone; $A_{st}$—section area of tensile reinforcement; $\alpha_{c1}$ and $\beta_{c1}$—characteristic parameters of equivalent rectangular stress pattern about RAC; $f_{cc}$—axial compressive strength of RAC; $f_{y}$—yield strength of the reinforcement; $f_{ut}$—tensile strength of UHPC; and $M_{u}$—ultimate bending moment of the combined section.

Xiao et al. [109] introduced an innovative “graded recycled aggregate concrete (GRAC) slab” design concept, fabricating specimens with gradient recycled aggregate replacement ratios (0%, 50%, 100%) across the cross-section. The loading method is four-point loading. Studies revealed that the flexural deformation performance of GRAC slabs matched that of conventional concrete slabs, with flexural capacity and stiffness significantly improving with increasing reinforcement ratios. At reinforcement ratios of 0.70% and 0.98%, GRAC slabs exhibited satisfactory load-bearing capacity, and rational gradient design effectively enhanced the flexural performance of RAC slabs.

Luo et al. [110] proposed a semi-recycled and semi-normal concrete composite slab and conducted static flexural and double-sided shear tests to systematically compare its mechanical performance with conventional concrete members. The experimental loading method involves applying concentrated loads at two quarter-point positions. The results showed that the composite slab incorporating steel fiber-reinforced recycled aggregate concrete exhibited similar three-stage failure characteristics to normal concrete specimens, with full development of deflection and satisfactory ductility. Its load-bearing capacity met the requirements of civil building load specifications, and no cracks or slippage occurred at the composite interface, indicating collaborative work performance compliant with shear strength standards.

Zhu et al. [111] investigated the flexural mechanical properties of RAC composite slabs. The interface has been roughened. The experimental loading method is three-point symmetric loading. The experiment found that their mechanical performance was comparable to conventional concrete members. A correction factor for the short-term stiffness calculation formula has been proposed.

Zhou et al. [112] studied the mechanical behavior of RAC slabs, revealing that both cracking and ultimate loads were inversely proportional to the recycled aggregate replacement ratio, with a more significant reduction in ultimate load. A correction coefficient for the RAC slab calculation formula was also proposed.

**Table 7:** Integration of RAC research results.

Component Type	Component Style	Style Source	Parameter Settings	Dimension	Research Results
Composite beam	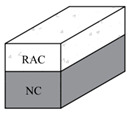	Reference [104]	Composite beam shear-span ratio (1.3, 1.6, 1.9, 2.2)	240 × 120 mm	1. The ultimate loads of RAC composite beams are 129.0 kN, 121.0 kN, 94.5 kN, and 81.2 kN. The ultimate loads of the corresponding RC composite beams are 122.8 kN, 111.0 kN, 92.8 kN, and 78.5 kN. 2. The failure mode of recycled concrete composite beams is similar to that of ordinary concrete composite beams. 3. The ultimate bearing capacity decreases with the increase in shear-span ratio.
	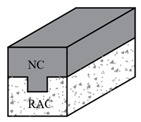	Reference [105]	Section combination form (C-type and U-shaped) and Shear span-to-depth ratio (1.5, 2.0, 3.0)	400 × 200 mm	1. As the shear span-to-depth ratio increases, the loads on the C-type composite beam are 420.8 kN, 350.4 kN, and 255.6 kN. The loads of the U-type composite beam are 623.9 kN, 192.3 kN, and 221.0 kN. The RC beam (3.0) is 150.9 kN. 2. The C-type and U-shaped composite beams with a shear-span ratio of 3.0 have good load-bearing performance, just like the cast-in-place beams. 3. The composite beam has not experienced any adverse effects on its bearing capacity and deformation.
	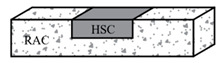	Reference [106]	Composite beam material composition (NC, RAC, RAC-HSC, HSC)	200 × 120 mm	1. According to different materials, the average critical loads of the experimental beams are 82.25 kN, 77.7 kN, 104.6 kN, and 98.4 kN. 2. The bending capacity of composite beams is 20% to 36% higher than that of cast-in-place recycled concrete beams. 3. The deflection is reduced by 20% to 40%, resulting in higher stiffness and bending resistance.
	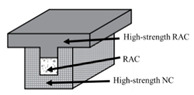	Reference [107]	Composite beam material composition (HSC, HSRAC, NSRAC, HSRAC-NSRA)	630 × 500/250 mm	1. According to different materials, the ultimate loads of the experimental beams are 317.5 kN, 361.3 kN, 317.5 kN, and 348.8 kN. The deflection is 126.4 mm, 129.0 mm, 67.4 mm, and 110.0 mm. 2. The bending strength and yield load of the composite beam have been improved compared to the reference beam, but the ductility has decreased. 3. The deformation capacity of ordinary-strength recycled concrete filled beams is consistent with the reference beams, and the compressive strength is similar.
Composite slab	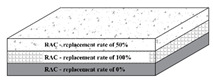	Reference [109]	Gradient distribution RAC replacement rate (0-100-50, 50, 50-100-0), longitudinal reinforcement ratio (0.42, 0.70, 0.98)	90 × 400 mm	1. When the substitution rate is 0-100-50, as the longitudinal reinforcement ratio increases, the ultimate loads are 13.2 kN, 31.6 kN, and 39.6 kN. When the substitution rate is 50, the ultimate loads are 19.5 kN, 30.5 kN, and 39.2 kN. When the substitution rate is 50-100-0, the ultimate loads are 17.7 kN, 29.3 kN, and 39.8 kN. 2. The bending deformation performance of gradient slabs is comparable to that of ordinary concrete slabs, and their bending bearing capacity and stiffness are significantly enhanced with the increase in reinforcement ratio. 3. When the reinforcement ratio is 0.70% and 0.98%, the gradient plate exhibits good bearing capacity.
	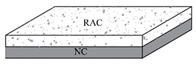	Reference [111]	Reinforcement ratio (0.51, 1.01, 1.52)	160 × 1000 mm	1. As the reinforcement ratio increases, the ultimate loads of RAC-NC composite slabs are 67.7 kN, 139.6 kN, and 174.0 kN. The ultimate load of the full RAC composite slab (1.01) is 135.1 kN. 2. The composite panels of recycled concrete have similar three-stage failure characteristics to ordinary concrete specimens. 3. The mechanical properties are similar to those of ordinary concrete components.

Research on RAC and its application in structural members has made certain progress, and studies have shown that recycled aggregate concrete can be used in composite components. However, there are still some deficiencies in stiffness, bearing capacity calculation, and other aspects. Subsequent research can focus on addressing the deficiencies of RAC to enable its better utilization.

## 4. Challenges and Suggested Improvements

(1) In the current research on composite concrete structures for flexural members, studies on beam components have outpaced those on slab components, resulting in a relatively mature design theory for beams. However, research on slab components lags significantly behind, with limited existing achievements. This research imbalance has directly led to deficiencies in the design theories and methodologies for composite concrete slabs.
(2) While composite concrete structures, which combine different concrete types, demonstrate significant advantages in material performance optimization and structural efficiency, their engineering applications remain limited. Current experimental and numerical studies predominantly focus on short-term mechanical properties (e.g., flexural capacity, shear strength), yet long-term monitoring data on durability (e.g., chloride ion erosion, carbonation depth), fatigue performance, and creep effects under complex environmental conditions are scarce.
(3) The existing design codes are predominantly based on single-material systems and inadequately address the nonlinear behaviors of multi-material composite structures, such as differential shrinkage/creep effects and temperature stress distribution. Under complex loading scenarios (e.g., earthquakes, impacts), the failure modes of composite structures may deviate from theoretical predictions.
(4) One of the improvements suggested in subsequent research is to conduct studies on the dynamic performance (such as explosion resistance and impact resistance) and long-term fatigue performance of composite components, as these properties are of great significance for buildings in specific working environments.
(5) Another recommendation in subsequent research is to conduct durability studies on composite components under extreme environments, aiming to accumulate data on the relevant performance degradation of composite components in such conditions and provide a basis for the durability design of these components.
(6) Furthermore, the specifications currently relied upon in the design of composite components are mostly standardized norms tailored for single materials. In the future, research efforts should be expedited to focus specifically on the theoretical framework for composite component design. It is essential to propose calculation formulas and design theories applicable to different material combinations and cross-sectional forms, and to formulate standardized specifications that cater to composite components.

## 5. Conclusions

This paper reviews some studies on flexural members in composite concrete structures, with the following main conclusions:

(1) UHPC exhibits significant advantages in engineering applications with extreme load and durability requirements due to its ultra-high mechanical strength, excellent toughness, superior durability, and unique microcrack self-healing properties. However, its large-scale engineering application is restricted by the notable hydration heat effect caused by high cement content, the shrinkage cracking tendency induced by complex production processes, and the high manufacturing cost. Although ECC demonstrates excellent mechanical properties in compression, tension, and bending, as well as superior long-term durability, its large drying shrinkage strain and relatively low elastic modulus impose higher requirements on structural deformation control. RAC aligns with China’s “dual carbon” strategic goals by optimizing costs through the recycling of construction waste. However, its inherent defects, such as high aggregate crushing index, insufficient density, and high water absorption, result in a gap in workability and durability compared to natural aggregate concrete.
(2) Through collaborative design at the material, component, and structural levels, composite concrete structures can achieve comprehensive benefits of reduced cost and enhanced performance while ensuring safety. This approach breaks through the limitations of single materials, with UHPC enhancing structural strength, ECC improving structural crack resistance, and RAC rendering structures more economical and environmentally beneficial.
(3) The interfacial treatment method significantly affects the interfacial bond strength of composite components. Experimental evidence indicates that interfacial bond strength increases with higher surface roughness. For instance, Reference [65] demonstrated that mechanical treatments such as grooving and drilling achieved approximately 35% higher bond strength compared to wire brushing methods. Similarly, Reference [66] reported that sand blasting or drilling treatments, which create high surface roughness at the interface, resulted in 30–40% greater bond strength than untreated interfaces.
(4) The structural load-bearing capacity of composite components is closely related to the thickness distribution of constituent materials within the cross-section. A strategic increase in UHPC or ECC layer thickness can significantly enhance the ultimate load-bearing capacity of composite components. For instance, Reference [57] reported that selecting an appropriate UHPC layer thickness could achieve an approximate 40% improvement in ultimate load capacity compared to NC beams. Similarly, Reference [93] demonstrated that incremental ECC layer thickness leads to 5–25% enhancement in ultimate bearing capacity.
(5) The modification of composite configuration can influence the mechanical properties of composite components to a certain extent. For instance, Reference [60] reported that positioning the UHPC layer at the bottom compromises ductility, while locating it at the top enhances ductility. Reference [93] demonstrated that composite beams with U-shaped cross-sections exhibit 10–20% higher ultimate load-carrying capacity compared to those with rectangular cross-sections. In future research on composite slabs, considering that composite slabs typically have a large width, improvements can be made to the U-shaped section by adding longitudinal ribs internally. This modification aims to further enhance the overall synergistic performance of the composite slab structure.
(6) The material composition of composite components significantly influences their exhibited mechanical properties. For instance, Reference [75] indicates that as the steel fiber content in UHPC increases, the ultimate displacement of members decreases accordingly. Reference [109] reports that while increasing the longitudinal reinforcement ratio leads to elevated load-bearing capacity, varying replacement ratios of recycled aggregate may either enhance or diminish this capacity, depending on specific formulation parameters.
(7) The mechanical performance of composite components is significantly affected by different loading configurations. Under four-point bending loading, the composite plate exhibits the highest load-bearing capacity, whereas single-point eccentric loading represents the most unfavorable loading condition, resulting in relatively lower load-bearing capacity. Eccentric loading scenarios should be strictly avoided in practical engineering applications.

## Figures and Tables

**Table 1:** Abbreviations of professional nouns.

Abbreviations of Professional Nouns	
Ultra-high-performance concrete	UHPC
Ductile Fiber-Reinforced Cementitious Composite	DFRCC
Engineered cementitious composite	ECC
Fiber-reinforced polymer	FRP
Graded recycled aggregate concrete	GRAC
High-Performance Concrete	HPC
High-strength concrete	HSC
Normal concrete	NC
Recycled aggregate concrete	RAC
Reinforced concrete	RC
Strain-Hardening Cement-Based Composite	SHCC
Seawater sea-sand engineered cementitious composite	SS-ECC
Ultra-High Toughness Cementitious Composite	UHTCC

**Table 2:** Comparison of performance data of cementitious materials.

Performance Indicators	Compressive Strength (MPa)	Flexural Strength (MPa)	Elastic Modulus (GPa)	Fracture Energy (kJ/m^2^)	Chloride Ion Diffusion Coefficient (10^–12^ m^2^/s)	Carbonation Depth (g/cm^2^)	Apparent Density (kg/m^3^)
UHPC	120~200	12~60	40~60	10~20	<0.02	<0.5	2400~2800
ECC	50~160	10~25	20~60	2~10	<0.1	<1.5	1800~2200
NC	20~50	2~5	<35	0.12	1.1	>10	2200~2600

**Table 3:** The shear properties of UHPC-NC and NC-NC bonding.

References	Test Method	Interface Treatment Method	UHPC-NC (MPa)	Failure Mode	NC-NC (MPa)	Failure Mode
ACI specification recommended value [63]	Simple shear		1 day 1.0–2.1 7 day 2.1–2.8 28 day 2.8–4.1			
	Oblique shear		1 day 2.8–6.9 7 day 6.9–12 28 day 14–21			
[64]	20° Oblique shear	High-pressure water injection	9.4–18.1	C	9.2–15.3	A or B
	25° Oblique shear		10.9–26.9	C	10.4–18.5	A or B
	30° Oblique shear		12.9–28.8	C	11.7–22.3	A or B
[65]	30° Oblique shear	Wire brush	18.58	B	13.46	B
		Drill	25.10	C		
		Carving groove	25.39	C		
[66]	Oblique shear	Not processed	15.83–20.29	A		
		Drill	20.35–22.45	B		
		Sand blast	27.24–28.81	B		
	Twin shear	Not processed	3.47–4.90	A		
		Drill	6.39–7.02	C		
		Sand blast	3.48–5.24	C		
[67]	Simple shear		1.86–21.76	A or B	1.6–12.64	A or B

Note: In Table 3, the letters A, B, and C denote three distinct failure modes: A represents pure bonding surface damage, while UHPC and NC remain intact without obvious damage; B represents the failure of the bonding surface and cracking of the NC matrix concrete; C represents the destruction of the bonding surface matrix.

**Table 4:** Experimental design and results of UHPC composite components.

References	Parameter Settings	Variable	Component Style	Dimension	Experimental Result
[57]	UHPC thickness	0 mm	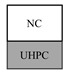	400 × 200 mm	1. The bearing capacity increases with thickness and is 311.24 kN, 296.18 kN, 352.66 kN, 435.49 kN, 454.31 kN, respectively. Increasing the thickness of UHPC can improve the load-bearing capacity of components. 2. The midspan deflection is 34.21 mm, 23.66 mm, 4.49 mm, 6.67 mm, 6.61 mm. Increasing the thickness of UHPC can suppress component cracking.
		100 mm			
		150 mm			
		200 mm			
		400 mm			
[58]	Reinforcement method	Unreinforced RC	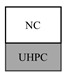	400 × 200 mm	1. The ultimate loads are 289.5 kN, 407.0 kN, and 529.4 kN, respectively. The flexural performance of RC beams is significantly improved after being reinforced with UHPC. 2. When the crack width is 0.05 mm, the load is 66.5 kN, 115.0 kN, and 170 kN; when the width is 0.10, the load is 95.6 kN, 190.8 kN, and 280.5 kN; when the width is 0.2 mm, the load is 139.3 kN, 325.7 kN, and 430.2 kN. The reinforced beam has a significant improvement in the load of characteristic joint width at all levels compared to the untreated beam.
		Conventional reinforcement with UHPC		450 × 200 mm	
		Prestressed UHPC reinforcement			
[60]	UHPC thickness and location	NC	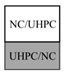	250 × 150 mm	1. The ultimate loads are 140.58 kN, 195.66 kN, 169.18 kN, 178.47 kN, 162.79 kN, and 164.31 kN, respectively. Placing UHPC at the bottom of the beam has a positive effect on improving the cracking load, yield load, and ultimate load of the composite beam. 2. The ductility is 3.86, 4.14, 3.07, 3.43, 4.98, and 4.9, respectively. Placing UHPC at the bottom of the beam will weaken the ductility of the composite beam, while placing it at the top of the beam can improve ductility.
		UHPC			
		Bottom 50 mm			
		Bottom 100 mm			
		Top 50 mm			
		Top 100 mm			
[62]	U-shaped UHPC template		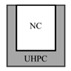	1120 × 900 mm	The new composite cover beam has excellent bending and shear resistance, with a bending bearing capacity of 12,285.5 kN·m and a shear bearing capacity of 4459.2 kN.
[72]	Interface processing methods	Natural vibration	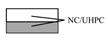	500 × 150 mm	1. The results demonstrated that conventional approaches (including natural vibration, surface troweling, roughening, and shear reinforcement configuration) effectively prevented shear failure at the composite interface prior to flexural failure.
		Smoothing			
		Roughening			
		Installation of shear-resistant steel bars			
		Ordinary concrete roughening			
[75]	UHPC thickness (mm)	0, 50, 80, 100, 200	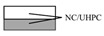	600 × 200 mm	1. The ultimate loads of composite panels with different thicknesses of UHPC are 440 kN, 440 kN, 460 kN, 470 kN, and 655 kN, respectively. 2. The ultimate loads of composite panels with different reinforcement ratios (1.57%, 1.63%) are 350 kN and 460 kN, respectively. 3. The ultimate loads for different steel fiber volume fractions (1.5%, 2%) are 455 kN and 460 kN, respectively. The ultimate displacements are 91.67 mm and 61.15 mm, respectively.
	Reinforcement ratio	1.57%, 1.63%, 3.14%			
	Steel fiber content	0%, 1.5%, 2%, 3%			

**Table 5:** Summary of the interfacial bond strength between ECC and ordinary concrete.

Reference	Specimen Series Number	Bond Strength/MPa				Details
		Slant Shear Test	Direct Shear Test	Double-Sided Direct Shear Tests	Splitting Tensile Test	
Wei et al. [89]	EP			3.72		Point grooves
	ER			2.15		Rectangular grooves
Wu et al. [90]	P-50-ER		2.35			Precast and epoxy resin, ECC strength is 50 MPa
	P-80-ER		2.37			Precast and bolt, ECC strength is 80 MPa
	C-50-B		1.83			Casting and bolt, ECC strength is 50 MPa
	C-80-B		2.03			Casting and bolt, ECC strength is 80 MPa
Qasim et al. [91]	PVA-AC	3.98			3.94	SPH-ECC, As-cast
	PVA-SB	4.91			4.68	SPH-ECC, Sandblast
	SPH-AC	3.86			3.85	PVA-ECC, As-cast
	SPH-SB	4.78			4.21	PVA-ECC, Sandblast
Jiang et al. [92]	TNII.E				2.45	“N”, “P”, and “S” represent no interfacial agent, cement paste interfacial agent, and polymer-modified interfacial agent, respectively. The chiseled interfaces are labeled as I, II, III, and the groove interface is IV
	TPII.E				2.68	
	TSII.E				2.19	
	VNI.E		2.50			
	VNII.E		3.19			
	VNIII.E		3.52			
	VNIV.E		3.45			
	VPII.E		3.62			
	VSII.E		2.50			

**Table 6:** Experimental design and results of ECC composite components.

References	Parameter Settings	Variable	Component Style	Dimension	Experimental Result
[82]	Interface processing methods	Not handled	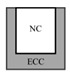	150 × 120 mm	1. The ultimate loads of the composite beam and RC beam with three interface processing methods are 84.6 kN, 87.1 kN, 87.2 kN, and 75.7 kN, respectively. 2. The ductility of composite beams and RC beams with three interface treatment methods is 2.74, 3.43, 3.36, and 2.79, respectively. 3. Different interface treatments have a relatively small impact on the bearing capacity of composite beams but have a significant impact on the ductility coefficient. The ductility coefficients of the untreated composite beam and RC beam are basically the same, indicating that good bonding between ECC and concrete can effectively increase the ductility of the component.
		Place coarse sand			
		Horizontal groove			
		RC beam			
[83]	Thickness of overlay layer and strengthening of overlay layer	Additional layer thickness: 60 mm, 90 mm	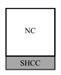	230 (260) × 150 mm	1. The ultimate loads of the 60 mm and 90 mm SHCC layers are 121 kN and 128.2 kN, respectively. The ultimate loads of the reinforced SHCC layer are 161.05 kN and 179 kN, respectively. The corresponding ultimate loads of the RC beam are 118 kN, 127.5 kN, 146 kN, and 152.79 kN. The SHCC overlays increased the stiffness and load-carrying capacity of the SHCC-RC overlay beams compared to the control RC beams with a concrete overlay.
		Strengthen the thickness of the overlay layer: 60 mm, 90 mm			
		RC beams with the same geometric shape			
[93]	ECC thickness (mm), steel bar diameter (S), and ECC layer shape	0-S-14	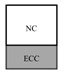 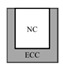	300 × 200 mm	1. The ultimate loads of composite beams with different ECC layer thicknesses are as follows: 211.1 kN, 221.8 kN, 225.2 kN, 236.8 kN, 263.4 kN. 2. The ultimate loads of composite beams with different reinforcement ratios are as follows: 149.9 kN, 225.2 kN, and 333.9 kN. 3. The ultimate loads of U-shaped composite beams and reference beams (0 mm-S-14, 30 mm-S-14) are 241.49 kN, 211.1 kN, and 221.8 Kn.
		30-S-14			
		60-10/14/18			
		90-S-14			
		120-S-14			
		U-shape-S-14			
[32]	ECC thickness and electricity corrosion time	0 mm, 40 mm	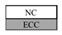	100 × 500 mm	1. The ultimate loads of RC slab and electrified corrosion for 15 days and 45 days are, respectively, 46.6 kN, 52.7 kN, and 41.4 kN. 2. The ultimate loads of PVA-ECC slab and electrified corrosion for 15 days and 45 days are 50.2 kN, 54.6 kN, and 52.8 kN, respectively. 3. Under the same corrosion time, comparing RC slab with PVA-ECC slab, the ultimate load of PVA-ECC slab is improved, and its bending resistance is stronger.
		Electrified and corroded for 0 days, 15 days, and 45 days.			
[99]	Loading form	Four-point bending loading	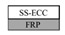	100 × 600 mm	1. Four-point loading limit load: 206.8 kN. Three-point loading limit load: 180.0 kN. Limit loads at different positions are 169.4 kN, 144.6 kN, 151.8 kN, and 106.3 kN. 2. The load-bearing capacity of the composite slab under the four-point bending was the highest, whereas that under single-point loading was significantly lower. Single-point eccentric loading was a more unfavorable working condition for the composite slab, particularly for the specimen with eccentric loading points in both the transverse and longitudinal directions, which had the smallest bending deflection and energy absorption.
		Three-point bending loading			
		Central or eccentric single-point load at different positions.			
[100]	Precast plank thickness	40 mm, 60 mm	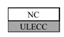	120 (160) × 500 mm	1. The ultimate loads of 60 mm RC slab (2I) and ULECC slab (3II) are 92.2 kN and 172.4 kN. 2. The ultimate loads of 60 mm ECC composite slab (2I, 3I, 3II) are 118.8 kN, 110.4 kN, and 159.1 kN. The ultimate load of 40 mm ECC composite slab (3I) is 105.8 kN. 3. The bearing capacity of ECC composite slab is improved by 29%, and its own weight is reduced by 24%.
	Lattice girder	2 type-I, 3 type-I, 3 type-II			
	Overall slab height	H = 120, 160 mm			

## Data Availability

No new data were created or analyzed in this study. Data sharing is not applicable to this article.
